# Overview of cardiac toxicity from radiation therapy

**DOI:** 10.1111/1754-9485.13757

**Published:** 2024-09-20

**Authors:** Vicky Chin, Robert N Finnegan, Paul Keall, James Otton, Geoff P Delaney, Shalini K Vinod

**Affiliations:** ^1^ Department of Radiation Oncology Liverpool and Macarthur Cancer Therapy Centres Sydney New South Wales Australia; ^2^ Image X Institute University of Sydney Sydney New South Wales Australia; ^3^ South Western Sydney Clinical School University of New South Wales Sydney New South Wales Australia; ^4^ Ingham Institute for Applied Medical Research Sydney New South Wales Australia; ^5^ Northern Sydney Cancer Centre Royal North Shore Hospital Sydney New South Wales Australia; ^6^ Institute of Medical Physics University of Sydney Sydney New South Wales Australia; ^7^ Department of Cardiology Liverpool Hospital Sydney New South Wales Australia

**Keywords:** cardiac substructures, cardiac toxicity, heart dose, thoracic radiotherapy

## Abstract

Radiotherapy is an essential part of treatment for many patients with thoracic cancers. However, proximity of the heart to tumour targets can lead to cardiac side effects, with studies demonstrating link between cardiac radiation dose and adverse outcomes. Although reducing cardiac dose can reduce associated risks, most cardiac constraint recommendations in clinical use are generally based on dose to the whole heart, as dose assessment at cardiac substructure levels on individual patients has been limited historically. Furthermore, estimation of an individual's cardiac risk is complex and multifactorial, which includes radiation dose alongside baseline risk factors, and the impact of systemic therapies. This review gives an overview of the epidemiological impact of cancer and cardiac disease, risk factors contributing to radiation‐related cardiotoxicity, the evidence for cardiac side effects and future directions in cardiotoxicity research. A better understanding of the interactions between risk factors, balancing treatment benefit versus toxicity and the ongoing management of cardiac risk is essential for optimal clinical care. The emerging field of cardio‐oncology is thus a multidisciplinary collaborative effort to enable better understanding of cardiac risks and outcomes for better‐informed patient management decisions.

## The impact of cancer and cardiovascular disease

Cancer survival has steadily increased through the years, with radiotherapy being an essential part of treatment for many malignancies.^1^, ^2^, ^3^, ^4^ With improvements in long‐term cancer survival, preventing and managing potential toxicities is a priority to ensure best outcomes and quality of life for patients. In thoracic radiotherapy, which is commonly used for breast, lung, oesophageal cancers and lymphoma, cardiac side effects may occur due to the proximity of the heart to target volumes. It is therefore imperative to understand the impact of radiation on the heart, the radiosensitivity of different cardiac substructures and contributions from factors such as baseline cardiac comorbidities, as well as chemotherapeutic and immunological agents. This will aid in optimising and personalising radiotherapy plans, in order to minimise cardiac toxicities.

Cancer and cardiovascular diseases are two of the top five burdens of disease in Australia.^5^ Cancer is the top‐ranked burden, with an estimated 165,000 new cancer diagnoses each year.^1^ Thoracic cancers contribute one of the highest numbers: breast cancer, the second most diagnosed malignancy in Australia, accounted for an estimated 20,640 new cases in 2022.^6^ Lung cancer, the fifth most common malignancy (and second most common thoracic malignancy after breast cancer) contributed an estimated 14,529 new cases in the same year.^7^ As cancer treatments continue to evolve, cure rates are improving. For breast cancer, the 5‐year relative survival has improved from 78% in 1990–1994, to 92% in 2015–2019.^1^ In 2017, it was estimated that there were 79,720 people living in Australia who were diagnosed with breast cancer within the previous 5 years, and 243,539 living who were diagnosed within the previous 36 years.^6^ This figure is expected to grow, given the increasing incidence and survival of breast cancer.^6^ Even in lung cancer, where late toxicities were traditionally perceived as less important due to poorer survival rates, long‐term survival is rising.^7^ Australian data indicated that in 2017 there were 21,765 people living with lung cancer diagnosed within the previous 5 years, and 34,171 living who were diagnosed within the previous 36 years.^7^ These numbers will likely increase with the use of stereotactic radiotherapy for early‐stage disease and immunotherapy in locally advanced disease, both of which improve overall survival.^8^, ^9^, ^10^, ^11^ Given the high indications for use of radiotherapy in these cohorts,^2^ the number of patients who may potentially be impacted by treatment‐related cardiac conditions is significant. Thus, the ability to accurately predict the likelihood of survival versus cardiac toxicities from treatment is paramount to optimise individualised therapy.

Cardiovascular disease is also amongst the top five burdens of disease in Australia, with 1.2 million Australians living with a condition related to the cardiovascular system.^5^, ^12^ In Australia, cardiovascular diseases accounted for 25% of all deaths (42,700 people) in 2021, ranking second behind cancer deaths (which accounted for 30% of all deaths).^12^ Of these, 41% was due to coronary heart disease, 20% from stroke and 11% due to heart failure and cardiomyopathy. Worldwide, it is the leading cause of death, accounting for 32% (17.9 million) of all deaths in 2019.^13^ Not only is the mortality high, there were also 600,000 hospitalisations attributable to cardiovascular disease as the principal diagnosis in Australia in 2020–2021.^12^ The majority of the hospitalisations (27%) were due to coronary heart disease, followed by atrial fibrillation (13%) and heart failure and cardiomyopathy (12%). Coronary heart disease was the number one ranked disease‐specific burden in Australia, with an estimated 571,000 Australians living with the condition.^5^, ^14^

As many cancer treatments can impact cardiovascular health, strategies to minimise cardiovascular side effects of treatment, as well as monitoring and managing toxicities, are crucial. However, it is difficult to quantify cardiac risk from treatment in an individual patient. This is because cardiac risk is multifactorial, dependent not only on radiotherapy dosimetry, but also an individual's baseline cardiac risk factors and cardiotoxic systemic therapies. A collaborative effort is therefore required to better understand the complex effects of radiation on the heart and to better inform patients regarding likely treatment benefit versus potential side effects, in order to achieve improved patient outcomes.

## Factors contributing to cardiac toxicity in radiation therapy

Various patient, tumour and treatment factors contribute to the likelihood of cardiotoxicity in thoracic radiotherapy for malignancies of breast, lung, oesophagus and lymphoma. These are summarised in Table 1.

**Table 1:** Patient, tumour and treatment factors contributing to cardiac toxicity in radiation treatment

Patient	Tumour	Treatment
Age ‐ Radiation effects may have greater impact on those treated at a younger age, though older age is associated with higher likelihood of cardiac comorbidities Gender Family history of cardiac disease Personal history of cardiac conditions, for example ischaemic heart disease, congestive cardiac failure and arrhythmias Hypertension Hypercholesterolaemia Diabetes mellitus Smoking history	Target volume location and proximity to the heart Target volume size Stage of cancer Prognosis of cancer	Systemic therapies which may also have cardiac side effects Total radiation dose, dose per fraction of radiation and volume of heart receiving radiation dose The use of radiotherapy techniques to reduce cardiac dose, for example deep inspiratory breath hold and prone breast radiotherapy Previous thoracic radiotherapy and cumulative cardiac doses in re‐treatment Image guidance Treatment with proton or heavy ion therapy

### Patient factors

Baseline patient characteristics are prognostic for potential cardiac side effects. Young age at time of treatment may increase risks of long‐term cardiac side effects.^15^, ^16^ The presence of pre‐existing cardiac risk factors and comorbidities, such as history of ischaemic heart disease, hypertension, diabetes and family history, also elevate an individual's baseline risk. There are cardiac risk estimation tools used internationally such as the American Framingham risk equation,^17^, ^18^ the Systematic Coronary Risk Evaluation (SCORE) from Europe^19^ and QRISK/QRISK2 developed in the UK.^20^, ^21^ These models were generally developed from local population data and may not be generalisable, particularly to oncology patients, who often possess additional cardiac risks from their cancer treatments.

Patient factors are often intertwined with tumour subtypes, as demographics for these cancers tend to differ. Patients with Hodgkin's lymphoma and breast cancer tend to be younger with fewer comorbidities.^22^, ^23^, ^24^ Patients with lung and oesophageal cancer are usually older, more frequently males, and often have smoking‐related cardiac comorbidities.^22^, ^23^, ^24^ These factors pose challenges in evaluating cardiac morbidity and mortality, as it can be difficult to define whether a cardiac event is secondary to treatment, pre‐existing comorbidities or a combination, and whether cause of death is from malignancy or their comorbid condition.

### Tumour factors

Disease prognosis, with the expected treatment benefit versus potential side effects, needs to be carefully considered when evaluating cardiac toxicity risks. As patients with breast cancer and lymphoma generally have good long‐term survival, these groups were the focus of early cardiotoxicity studies. While the perception that late toxicities were of lesser importance in lung and oesophageal cancer, there is now evidence that cardiac toxicity can manifest within months of treatment^25^, ^26^, ^27^ and that physiological changes to the heart can be detected during a radiotherapy treatment course.^28^

Other factors that can impact cardiac dose include target volume, target location and disease stage. Larger tumours and advanced stage generally lead to bigger radiation target volumes and, if located adjacent to the heart, may lead to significant cardiac dose. A centrally located stage III lung cancer with mediastinal nodal involvement (compared to a small peripheral stage I lung cancer) or adjuvant radiotherapy for node‐positive breast cancer requiring internal mammary chain (IMC) treatment will likely result in higher doses to the heart. Irradiating the IMC can double the mean heart dose (MHD) compared to no IMC treatment.^29^ Radiotherapy to the left breast has also been associated with higher risk for coronary artery disease and cardiac death compared with the right breast.^30^

### Treatment factors

Treatment factors related to radiotherapy as well as systemic therapies must be considered. Various systemic agents utilised in the treatment of thoracic malignancies, such as anthracyclines, taxanes, cyclophosphamide and trastuzumab, have been implicated in cardiac toxicity and may be additive to the effects of radiotherapy, while investigations are ongoing for newer immunotherapy agents.^31^, ^32^, ^33^, ^34^

**Fig. 1:**
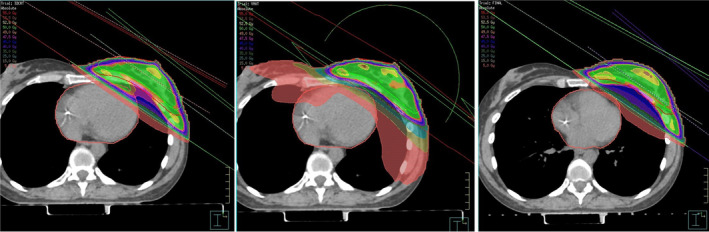
Example of left breast radiotherapy planned with different techniques. From left to right: Free‐breathing scan planned with 3D conformal tangent‐based technique; free‐breathing scan planned with volumetric modulated arc therapy (VMAT); deep inspiratory breath hold (DIBH) scan planned with tangent‐based technique. Note anterior part of heart can potentially receive high radiation dose with traditional tangent‐based technique, while larger volumes of low dose (5Gy salmon colour isodose) can be observed with VMAT technique. DIBH typically results in less dose to the heart by increasing distance between heart and anterior chest wall.

**Fig. 2:**
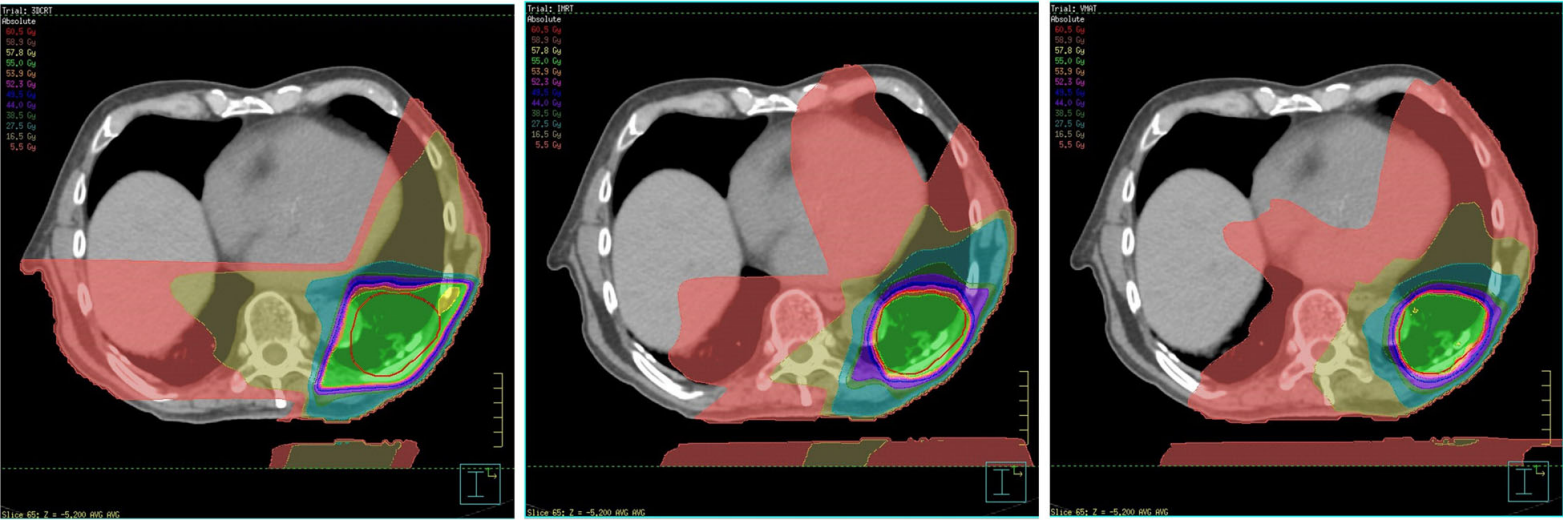
Example of lung radiotherapy planned with different techniques. From left to right: 3D conformal technique (3DCRT); intensity‐modulated radiotherapy (IMRT) and volumetric modulated arc therapy (VMAT). Note different dose distributions to the heart – in this example of 3DCRT, part of posterior heart receives 27.5Gy (cyan colour isodose), while IMRT/VMAT can improve conformality around target volume but with larger areas of low dose (5.5Gy salmon colour isodose) to the heart.

Intensity‐modulated radiotherapy (IMRT) and volumetric modulated arc therapy (VMAT) are now routinely used to deliver radiotherapy. The combination of inverse planning and dose conformality that can be achieved with these techniques allows increased target doses and reduced overall doses to organs at risk (OARs). However, these techniques fundamentally change typical dose distributions compared with historical field‐based or 3D conformal radiotherapy (3DCRT) from which many of the current planning constraints and risk models were derived. In a secondary analysis of the RTOG 0617, a trial investigating dose escalation in non‐small cell lung cancer, IMRT resulted in lower cardiac doses than 3DCRT, with no difference in survival.^35^ This is despite the IMRT group having more patients with advanced stage disease (stage IIIB) and larger treatment volumes. A single institution retrospective analysis by Speirs *et al*. also reported reduced cardiac doses with IMRT, as well as fewer post‐treatment cardiac toxicity events with this technique.^36^ An area of consideration for IMRT and VMAT is that while high‐dose areas may conform better to the target volume, plans exhibit increased ‘low‐dose wash’ covering nearby OARs. The impact of these low‐dose regions on long‐term cardiac outcomes is unclear and requires further investigation. Figures 1 and 2 demonstrate the variations in cardiac dose distribution that may be observed due to different radiation techniques for breast and lung cancer patients, respectively.

In addition to IMRT and VMAT, stereotactic ablative body radiotherapy (SABR) is now routinely used in patients with peripherally located medically inoperable stage I lung cancer and pulmonary oligometastases.^8^, ^37^, ^38^ There is increasing use of SABR for more central tumours in proximity to the heart, great vessels and proximal bronchial tree. This technique delivers high doses to the tumour while sparing OARs; however, the resulting steep dose gradients and differing biological effects of ablative hypo‐fractionation require increased attention, particularly as high doses may be delivered to parts of the heart, which could impair substructure function and impact survival.^39^, ^40^, ^41^

Deep inspiratory breath hold (DIBH) and prone patient positioning radiotherapy techniques can reduce cardiac doses. DIBH is often used in treatment of left‐sided breast cancers, as during inspiration the lungs expand, moving the anterior heart border posteriorly as the diaphragm moves inferiorly. Nissen *et al*. reported reduction in cardiac dose with this technique, with MHD approximately halved for left‐sided breast cancer DIBH plans compared with free‐breathing.^42^ Prone positioning is also used in some centres for breast cancer patients for whom heart dose may otherwise be high. The disadvantages of prone positioning include challenges with nodal treatment (requiring repositioning for nodal irradiation) and increased daily setup variation. Partial breast radiotherapy also has the potential to reduce dose to OARs such as the heart, in patients who are suitable for this technique.^43^, ^44^ Other methods to reduce cardiac dose include improvements in image guidance as well as proton therapy, an area of ongoing investigation.^24^, ^29^, ^45^ Even with such techniques, cardiac doses are inevitable when target volumes are located adjacent to the heart. Table 1 outlines some of the treatment factors to consider in regard to cardiac risks in thoracic radiotherapy.

## Overview of evidence in radiation‐induced cardiac toxicity

### Early studies and correlations with cardiac outcomes

Many early studies showing correlation between thoracic radiotherapy and cardiac risk were in breast cancer and lymphoma cohorts, given their expected prognosis and long‐term survival (Table 2). Studies of Hodgkin's lymphoma survivors identified associations between mediastinal radiotherapy and cardiac‐specific mortality, with young age at time of treatment being an important risk factor.^15^, ^16^, ^46^, ^47^ Long‐term cardiac morbidity is also a significant issue in this cohort.^31^, ^48^, ^49^ Aleman *et al*. reported that Hodgkin's survivors who received mediastinal radiotherapy had a twofold to sevenfold increase in risks of angina, myocardial infarction, heart failure and valvular disorders, at a median follow‐up of 18.7 years.^31^ The use of anthracycline chemotherapy further increased the risk of heart failure and valvular disorders, and those who received treatment before the age of 20 were at higher risk for angina and heart failure.^31^ However, studies of lymphoma patients were often based on older techniques, such as ‘mantle field’ radiotherapy, where large volumes of the mediastinum were within radiation fields. These older techniques are no longer used today, and cardiac morbidity and mortality from modern Hodgkin's treatment is presumed to be lower due to generally smaller fields of ‘involved site’ radiotherapy.

**Table 2:** Early studies and meta‐analyses showing correlation between cardiac outcomes and thoracic radiotherapy

Study	Cohort	Result	
Meta‐analysis Cheng *et al*. 2017^30^	Breast cancer 39 studies with 1,191,371 participants Years of radiotherapy treatment 1949–2008	Radiotherapy versus no radiotherapy:	Relative risk (RR):
		Coronary heart disease	RR 1.30, 95% CI 1.13–1.49
		Cardiac mortality	RR 1.38, 95% CI 1.18–1.62
		Left vs right breast:	
		Coronary heart disease	RR 1.29, 95% CI 1.13–1.48
		Cardiac mortality	RR 1.22, 95% CI 1.08–1.37
		Death from any cause	RR 1.05, 95% CI 1.01–1.10
Meta‐analysis Taylor *et al*. 2017^50^	Breast cancer 75 trials in 40,781 women Mean year of randomisation 1983 (interquartile range 1974–1989)	Radiotherapy versus no radiotherapy:	Rate ratio (RR):
		Cardiac mortality	RR 1.30, 95% CI 1.15–1.46
		Ischaemic heart disease	RR 1.31, 95% CI 1.13–1.53
		Heart failure	RR 1.94, 95% CI 1.27–2.98
		Valvular heart disease	RR 1.97, 95% CI 1.07–3.67
		Cardiac mortality excess rate ratio (ERR)	ERR per Grey mean heart dose (MHD):0.041
	Breast cancer Systematic review of lung and heart doses published 2010–2015 (median year irradiation 2010) 214 reports, 647 regimens ERR from historical trials above was combined with MHD from systematic review and population‐based cardiac mortality rates to give absolute risk estimates	Cardiac mortality absolute risk	Absolute risk: 1% for smokers 0.3% for non‐smokers
Van Nimwegen *et al*. 2015^49^	Hodgkin's lymphoma 2524 patients Years of radiotherapy treatment 1965–1995	Mediastinal radiotherapy increased risk of:	Hazard ratio (HR):
		Coronary heart disease	HR 2.7, 95% CI 2.0–3.7
		Heart failure	HR 2.7, 95% CI 1.6–4.8
		Valvular heart disease	HR 6.6, 95% CI 4.0–10.8
Boivin *et al*. 1992^46^	Hodgkin's lymphoma 4665 patients Years of diagnosis 1940–1985	Mediastinal radiotherapy increased risk of:	Relative risk (RR):
		Death from myocardial infarction	RR 2.56, 95% CI 1.11–5.93

In breast cancer cohorts, several meta‐analyses have shown correlation between radiotherapy and increased incidence of cardiac events and mortality. This includes a meta‐analysis of 39 studies, showing an increase in risk of coronary heart disease (relative risk RR 1.3, 95% CI 1.13–1.49), and cardiac mortality (RR 1.38, 95% CI 1.18–1.62) in those who had received breast irradiation compared to those who did not.^30^ The risk of coronary heart disease started to increase during the first decade, while cardiac mortality increased from the second decade post‐treatment.^30^ Increased risks were observed for left versus right breast irradiation, for coronary heart disease (RR 1.29, 95% CI 1.13–1.48), cardiac mortality (RR 1.22, 95% CI 1.08–1.37) and death from any cause (RR 1.05, 95% CI 1.01–1.10).^30^ A meta‐analysis by Taylor *et al*., with a total of 40,781 women from 75 trials that commenced prior to the year 2000 (median year of randomisation 1983), showed an increase in the risk of cardiac mortality post radiotherapy (rate ratio RR 1.30, 95% CI 1.15–1.46; *P* < 0.001) with an excess rate ratio (ERR) of 0.041 per Gy MHD.^50^ Combining the information from these early trials with MHD reported from publications dated 2010–2015 (median year of irradiation 2010) and population‐based cardiac mortality rates, it was estimated that the absolute risk of cardiac mortality was approximately 1% for smokers and 0.3% for non‐smokers.^50^ The estimations in this study were based on European population‐based cardiac mortality rates, and cardiac doses and radiation techniques utilised in the current era (such as IMRT and VMAT) are likely to differ from these earlier studies. The need to account for individualised baseline cardiac comorbidities, cardiac (and substructure) doses, and therefore personalised cardiac risk, will be important in future studies.

### Relationship between dose and outcomes: Whole heart dose

Despite correlations between thoracic radiation and cardiac toxicity, the relationship between cardiac dose and adverse outcomes remains unclear. Several studies have suggested cardiac dose thresholds – above which overall or cardiac‐specific mortality appear to increase. Hancock *et al*. studied 2232 patients with Hodgkin's lymphoma treated between 1960 and 1991 and showed that cardiac mortality risk increased for doses above 30Gy.^16^ Studies that specifically examined cardiac outcomes in children and adolescents with Hodgkin's lymphoma under 21 years of age, found similar dose thresholds. Castellino *et al*. found that the risk for overall mortality was associated with a radiation dose above 30Gy, while a second study by Hancock *et al*. found cardiac mortality risk increased above 40–45Gy.^15^, ^51^ The main limitation of these studies is the use of old techniques that are no longer used today.

Other studies have suggested a linear relationship between MHD and cardiac risk, rather than a threshold dose.^50^, ^52^, ^53^ As previously mentioned, for cardiac mortality, Taylor *et al*. showed an excess rate ratio (ERR) of 4.1% per Gy MHD in breast cancer radiotherapy.^50^ Darby *et al*. reported that the rate of major coronary events increases by 7.4% per Gy increase in MHD (95% CI 2.9–14.5).^52^ There was no threshold dose under which risk was not increased. Similarly, Van Nimwegen *et al*. demonstrated an excess relative risk of 7.4% per Gy of MHD (95% CI 3.3–14.8) for coronary heart disease in those who received mediastinal radiotherapy for Hodgkin's lymphoma.^53^ While this result appears to correlate with Darby *et al*.'s breast cancer study,^52^ the comparable patients in the van Nimwegen study (i.e. patients treated between ages 36 and 50), had an excess relative risk of 4.2% per Grey MHD (compared with Darby's 7.4% per Grey). These studies included treatments prior to the era of individual CT‐based 3D‐planning.

For lung cancer, studies have also supported an incremental relationship between MHD and cardiac events.^27^, ^54^ Wang *et al*. showed a 2‐year symptomatic cardiac events rate of 4% with MHD <10Gy, 7% with MHD 10–20Gy and 21% with MHD >20Gy.^27^ Cardiac events were defined as symptomatic pericardial effusion, acute coronary syndrome, pericarditis, significant arrhythmia and heart failure in this study.

Many of the above studies had focussed on reporting MHD as the main parameter; however, other studies suggest that perhaps it is the volume of the heart receiving a particular dose which affects outcomes, rather than the mean dose. RTOG 0617 suggested that heart V5 and V30 (the percentage of heart volume receiving >5Gy and >30Gy, respectively) were independent predictors of overall survival.^55^ The authors postulated that many of the recorded deaths may have been secondary to cardiac toxicity, though heart‐specific toxicity was not available in this trial to confirm this. Chun *et al*. and Speirs *et al*., on the other hand, identified that the whole heart V40 and V50, respectively, were significant factors for overall survival in their studies.^35^, ^36^ Table 3 summarises studies in support of different risk models based on whole heart dose parameters.

**Table 3:** Threshold versus linear versus volume‐based whole heart dose relationships

Cardiac parameter	Studies and outcome	
Threshold based	Hancock *et al*. 1993^16^	>30Gy increase cardiac mortality
	Hancock *et al*. 1993^15^	>40Gy increase cardiac mortality for age < 21
	Castellino *et al*. 2011^51^	>30Gy worse overall survival
Linear relationship rather than threshold dose	Darby *et al*. 2013^52^	ERR 7.4% per Gy MHD for major coronary events
	Van Nimwegen *et al*. 2016^53^	ERR 7.4% per Gy MHD for coronary heart disease
		ERR of 4.2% per Gy MHD for age 36‐50yo (for comparison with Darby *et al*.) for coronary heart disease
	Taylor *et al*. 2017^50^	ERR of 4.1% per Gy MHD for cardiac mortality
Volume‐based cardiac parameter	Bradley *et al*. 2015^55^	V5Gy V30Gy overall survival
	Chun *et al*. 2017^35^	V40Gy overall survival
	Speirs *et al*. 2017^36^	V50Gy overall survival

ERR, excess relative risk or excess rate ratio; MHD, mean heart dose.

To determine which cardiac dose parameters were significant for cardiac morbidity and survival, Zhang *et al*. performed a systematic review of 22 lung cancer studies with 5614 patients.^56^ Various cardiac dose‐volume parameters derived from the whole heart (e.g. MHD as well as volume‐based parameters such as V5), and anatomical sub‐regions (e.g. atria, ventricles) were included. In total, 94 unique cardiac dosimetric parameters were examined. While 20 parameters were found to be significant on multivariable analysis for overall survival or cardiac events in at least one study, no consistent relationship could be observed between any single dosimetric variable and outcomes. Limitations included the retrospective, single‐institutional design of many studies and heterogeneous endpoints. Several studies also included in excess of 20 cardiac dose‐volume parameters, and as many of these are highly correlated to one another, this may lead to imprecision in effect estimate of any individual dosimetric parameter. Speirs *et al*. demonstrated this in their study – while V50 was identified as the most important parameter, all heart parameters examined (heart maximum, minimum, mean doses and V5–V70 in 5Gy increments) significantly correlated with the heart V50.^36^ In addition to cardiac dosimetry, Zhang *et al*. were unable to successfully perform a meta‐analysis on the collated dosimetric and anatomic parameters, as most negative studies did not report effect estimates such as hazard ratios or odds ratios.^56^ Hence, despite this systematic review attempting to examine a comprehensive number of cardiac parameters, none were able to be identified as a significant predictor, with no good consensus on cardiac dose constraints, nor ‘safe’ dose below which there is no risk.

### Cardiac substructures: Cardiac‐specific endpoints and pathophysiology

Many of the previously described studies focussed on reporting doses to the whole heart. While intuitively, it makes sense that affecting a particular substructure may lead to specific corresponding endpoints, for example high doses to coronary arteries may lead to coronary artery disease and ischaemic events, information regarding dose constraints for cardiac substructures remains an area of ongoing investigation.

The pathophysiology of cardiac substructure‐specific outcomes, such as pericarditis, conduction disorders, coronary artery disease, valvular heart disease and cardiomyopathy, has been described in the literature.^23^, ^57^, ^58^, ^59^, ^60^, ^61^, ^62^ In brief, this is thought to be due to vascular damage and fibrosis, mediated by various acute and late‐acting inflammatory cytokines.^23^, ^57^, ^58^, ^59^, ^60^, ^61^, ^62^ In the case of pericarditis, the acute phase cytokines lead to increased vascular permeability, oedema and at times an effusion.^23^, ^57^, ^59^, ^60^, ^61^ Over time fibrotic reactions, thought to be mediated by cytokines such as transforming growth factor beta (TGF‐β), can lead to constrictive pericarditis, and chronic effusions can occur.^23^, ^57^, ^59^, ^60^, ^61^ Similar vascular damage and fibrosis also occur elsewhere in the heart, leading to other cardiac sequelae. Damage to endothelial cells and vessel walls affects both larger coronary arteries as well as the cardiac microvasculature.^23^, ^58^, ^59^, ^60^, ^61^ This vascular insufficiency can lead to myocardial ischaemia, along with diffuse fibrosis of the myocardial interstitium.^23^, ^58^, ^59^, ^60^, ^61^ Fibrosis may also occur within the cardiac conduction system and cardiac valves, leading to conduction disorders and valvular heart disease, respectively.^23^, ^58^, ^59^, ^60^, ^61^ These changes, or combination of these, affect both cardiac diastolic and systolic function, potentially leading to heart failure.^23^, ^59^, ^60^ Table 4 summarises the cardiac substructure‐specific toxicities, their pathophysiology and clinical cohorts at risk.

**Table 4:** Cardiac‐specific toxicities associated with thoracic radiotherapy

Toxicity	Timing	Pathophysiology	Regions of heart implicated	Clinical cohorts potentially at risk	Radiotherapy methods to reduce risk
Pericarditis Pericardial effusion Pericardial fibrosis	Acute‐Subacute (pericarditis and pericardial effusion) Long‐term (pericardial effusion and fibrosis)	Acute inflammation leads to increased vascular permeability, oedema and at times an effusion. Subsequent fibrosis can lead to constrictive pericarditis and chronic effusion	Pericardium	Radiation treatment with high doses to heart/pericardium, particularly where tumours are within or adjacent to mediastinum, for example oesophageal cancers, central lung or mediastinal tumours^27^, ^93^, ^94^, ^95^	IMRT/VMAT technique to avoid high doses to heart and pericardium DIBH may help reduce total volume of heart receiving radiation dose
Coronary artery disease	Long‐term	Damage to endothelial cells and coronary artery vessel wall, leading to atherosclerotic changes and stenosis of coronary arteries	Coronary arteries including the following: left main coronary artery (LMCA) left anterior descending artery (LAD) left circumflex artery (LCX) right coronary artery (RCA)	Coronary arteries receiving high dose depend on radiation site, for example, LAD in left breast treatment where anterior/apical parts of heart tend to receive high dose. This is in contrast to RCA in right breast radiotherapy^66^, ^67^ Radiation to base of the heart, for example when mediastinal lymph nodes are irradiated, may result in high dose to the origin of coronary arteries^26^, ^73^, ^74^ Increase in incidence of coronary artery disease was shown in Hodgkin lymphoma survivors where large volumes of the heart were within radiation field (mantle field radiotherapy)^46^, ^49^	DIBH: particularly in left‐sided breast radiotherapy or where IMC is included in target volumes Prone breast radiotherapy Consider omission of IMC treatment in breast cancer radiotherapy depending on likely benefit versus cardiac risk IMRT/VMAT technique to avoid high doses to coronary artery regions
Valvular heart disease	Long‐term	Fibrosis of cardiac valves	Aortic valve Pulmonary valve Mitral valve Tricuspid valve Evidence suggests left‐sided valves (aortic and mitral valves) are more likely to undergo radiation effects and stenosis^59^	Has been implicated in radiation treatments where large volumes of the heart (including cardiac valves) are within radiation field, such as mantle field radiotherapy^48^, ^49^	DIBH: may help reduce volume of heart, including valvular regions receiving radiation dose IMRT/VMAT technique to avoid high doses to heart and cardiac valves
Arrhythmia	Subacute Long‐term	Fibrosis of cells in conduction system leading to dysfunction and arrhythmia	Conduction system includes as follows: Conduction nodes (sinoatrial node and atrioventricular node) Conduction bundles and fibres However exact region affected by radiation leading to conduction system dysfunction is unclear	Radiation to base of heart has been postulated in the development of conduction disorders.^26^, ^39^, ^69^, ^73^, ^74^ This may be due to the heart base being related to structures such as sinoatrial node and pulmonary veins which have been implicated in the development of arrhythmias, in particular atrial fibrillation.^70^, ^72^ This is likely to be of particular importance in patients receiving mediastinal radiotherapy, for example locally advanced lung cancer, given the location of mediastinal nodes superior to the heart	IMRT/VMAT technique to avoid high doses to heart and particularly base of the heart region
Cardiomyopathy	Long‐term	Vascular insufficiency leading to myocardial ischaemia and fibrosis of myocardial interstitium	Myocardium	Unclear whether high doses to small area of myocardium or dose to large volumes of myocardium results in greater dysfunction. Cardiomyopathy due to myocardial fibrosis tends to lead to diastolic (rather than systolic) dysfunction^58^, ^59^ Coronary artery disease leading to myocardial ischaemia is another contributor for myocardial dysfunction	DIBH may help reduce total volume of heart receiving radiation dose Prone breast radiotherapy Consider omission of IMC treatment in breast cancer radiotherapy depending on likely benefit versus cardiac risk IMRT/VMAT technique to avoid high doses to the heart
Heart failure	Long‐term	Damage of various substructures and combination of these lead to diastolic and systolic dysfunction	Likely combination of aetiologies, for example ischaemic heart disease, valvular insufficiency, myocardial disease^58^, ^93^	Likely combination of dose and damage to various substructures Has been demonstrated in Hodgkin lymphoma survivors where large volumes of mediastinum would have received radiation dose, for example mantle field radiotherapy; as well as oesophageal cancer patients^49^, ^93^	DIBH may help reduce total volume of heart receiving radiation dose Prone breast radiotherapy Consider omission of IMC treatment in breast cancer radiotherapy depending on likely benefit versus cardiac risk IMRT/VMAT technique to avoid high doses to the heart

### Relationship between dose and outcomes: Cardiac substructures

The impact of cardiac substructure doses and their corresponding endpoints has been alluded to in early studies. Reconstructions of historical 2D planning data from the Hodgkin's lymphoma cohort showed potential detrimental impact of dose to cardiac valves and coronary arteries, for valvular heart disease and ischaemic events, respectively,^48^, ^63^ highlighting that mean heart dose may not be the only important (or predictive) cardiac dose‐volume parameter.

For breast cancer, several studies in the current 3D planning era have shown anterior and apical cardiac substructures to be at risk of higher radiation doses, with variable correlation of substructures doses with MHD. Van den Bogaard *et al*. found the LV‐V5 (volume of left ventricle receiving 5 Gy) to be an important prognostic parameter,^64^ while Tang *et al*., who further investigated LV doses utilising the American Heart Association 17 segment model,^65^ showed substantial radiation dose variation between different LV segments, with mid/apical anterior ventricular segments and apical region of LV receiving the highest dose.^66^ While there was correlation between MHD and both the entire LV and the anterior LV segments, correlation between MHD and doses to other cardiac substructures and LV segments was variable.^66^ Finnegan *et al*. showed that in a clinical trial cohort of 1,517 breast radiotherapy patients, the cardiac substructure receiving the highest mean dose in left‐sided treatment was the left anterior descending coronary artery (average ± standard deviation of 5.39 ± 3.46Gy), while the right coronary artery mean doses (1.92 ± 0.79Gy) were the highest in right‐sided treatment.^67^ Correlation was demonstrated between the MHD and mean doses to cardiac substructures in this clinical trial cohort of tangent‐based breast‐alone treatment. Jacob *et al*., on the other hand, analysed whole heart, LV and coronary artery doses of 104 patients treated with tangential breast 3DCRT (with or without regional nodal irradiation) from the BACCARAT study.^68^ In left‐sided breast cancer patients with MHD <3 Gy, LAD dose was above 40 Gy in 56% of patients, suggesting MHD may not be an adequate surrogate for substructure doses.^68^

In contrast, patients with lung cancer may receive higher cardiac doses overall, with heart region exposure highly dependent on tumour location and extent. Various cardiac regions have been implicated as significant, including dose to base of the heart, conduction system and coronary arteries.^23^, ^26^, ^56^, ^69^, ^70^, ^71^, ^72^ McWilliams *et al*., demonstrated that dose to the base of the heart, where the aorta, origin of the coronary arteries and sinoatrial node are located were associated with poorer survival.^26^ Contrary to RTOG 0617, the cardiac V5 and V30 were not significant for survival and nor was the mean heart dose,^26^ despite whole heart parameters being the focus of many early studies.^50^, ^52^, ^55^ Subsequent studies also support the cardiac base as an important region where dose may impact overall survival and risk of cardiac events.^69^, ^73^, ^74^ Regarding the conduction system, Kim *et al*. showed an association between maximum sinoatrial node dose with atrial fibrillation and overall survival in a cohort of 321 non‐small cell lung cancer and 239 small cell lung cancer patients,^70^ while Walls *et al*. suggested the possibility of pulmonary vein dose contributing to this arrhythmia endpoint.^72^ Atkins *et al*., however, suggested the importance of LAD V15 for major adverse cardiac events and mortality, with Mckenzie *et al*. supporting the importance of this dose parameter with significance for all‐cause mortality in a re‐analysis of the RTOG 0617 dataset.^71^, ^75^ Discordance between MHD and dose to LAD was also shown, with the whole heart parameter not being sufficient to predict LAD V15.^76^ Cardiac dose distributions in the lung cancer cohort are highly variable, which may be why MHD is less predictive for cardiac substructure doses in lung radiotherapy compared to breast cancer patients. Differences in the cardiac structures and dose parameters found to be significant between studies may be due to the multiple interactive factors that can influence heart dose, including target volume, target proximity to heart, as well as correlative nature of the whole heart volume and its substructures.^36^, ^56^, ^69^, ^73^, ^77^ As many existing lung cancer studies were retrospective with small case numbers or limited substructures, ongoing cardiac substructure toxicity studies are required to ascertain relationship between whole heart, substructure doses and correlation with cardiac outcomes.

Cardiac substructure doses and corresponding relationships with patient outcomes are of increasing relevance in the modern era of rapidly evolving radiation techniques. Dose distributions and treatment fractionations vary considerably between historical and modern radiotherapy techniques and although this has the potential to increase target doses and spare surrounding normal tissues, the impact of resulting changes to cardiac substructure exposure and resultant cardiac risk is unclear. Whole heart dose reporting, therefore, may not be adequate to understand and accurately determine the effects of radiation on the heart in this current radiotherapy era. The limitations of many existing cardiac substructure studies mean larger studies with a comprehensive set of cardiac substructures are required to further understand the impact of heart and substructure doses on patient outcomes for all thoracic tumour subsites.

## Role of automatic segmentation and considerations for cardiotoxicity studies

One of the challenges of large‐scale studies to investigate cardiac toxicity is the ability to delineate the heart and in particular, cardiac substructures, accurately and consistently on patient imaging. Manual contouring is labour‐intensive and time‐consuming, which limits the substructures and cases that are able to be feasibly contoured. Prior studies have included the use of standard or template anatomies to try and overcome this issue, but this may not account for individual anatomical and dose variations.^48^, ^52^, ^69^

Several cardiac substructure contouring atlases and guidelines have been published to aid in the definition of these volumes.^77^, ^78^, ^79^, ^80^, ^81^, ^82^ To assist with efficiency and consistency of structure delineation, automatic segmentation presents a potential solution, and various tools have been proposed.^83^, ^84^, ^85^, ^86^, ^87^, ^88^, ^89^ However, challenges deploying these tools at scale remain. Traditional atlas‐based auto‐segmentation techniques may be less accurate when anatomical variations are present, and while deep learning techniques have been shown to improve accuracy when compared to atlas‐based techniques,^86^, ^90^ training a deep learning model generally requires large, representative training datasets. As cardiac substructures are not yet routinely contoured in clinical practice, building sufficient datasets for deep learning modelling represents a significant barrier. Once an automatic segmentation tool has been developed, robust independent validation is also essential to ensure accuracy and consistency of segmentations.

Adding to this complexity is that uncertainties exist for the doses delivered to the heart. The heart is a dynamic organ – heart and substructure positions change with cardio‐respiratory motion, leading to variable doses delivered. Inter‐ and intra‐observer contouring variability has also been well described,^77^, ^79^, ^85^ and this is particularly relevant for structures that are difficult or cannot be seen on routine planning CT imaging such as coronary arteries, cardiac valves and conduction system. Adding a uniform margin to account for this may not be representative of the actual dose delivered. The magnitude of delineation variability is dependent on factors such as ease of structure delineation and non‐uniformity of cardio‐respiratory motion within and between patients.^91^, ^92^ The ability to account for these uncertainties, potentially with the assistance of automatic segmentation, will be useful in future cardiac toxicity research, as well as clinical implementation.

Validated automatic segmentation tools, with the ability to accurately delineate a comprehensive set of cardiac substructures, therefore has the potential to enable large‐scale studies. It can also provide consistency of delineation, potentially minimising issues encountered with inter‐observer variability. An ability to account for treatment uncertainties, such as motion and contour variations, will assist in understanding cardiac substructure doses received during treatment and is also an important consideration in the future of cardiac dose evaluation and plan optimisation.

## Conclusion and future directions

While there is clear evidence demonstrating a link between cardiac radiation dose and adverse outcomes, we still do not fully understand radiotherapy effects on the heart and substructures or have clear guidance on dose constraints. Quantifying an individual's cardiac risk remains challenging, as this needs to account for a patient's baseline cardiac risk, impact of systemic therapies, assessment of the risk‐benefit ratio for prescribing radiotherapy and modification of radiotherapy volumes or technique to lower cardiac toxicity risk.

Cardio‐oncology, an emerging multidisciplinary field enabling the collaborative effort required, plays an important role in the future of cardiotoxicity research. Large‐scale studies, with linkage of heart and substructure doses with cardiac‐specific outcomes are required to untangle the complex nature of cardiac radio‐sensitivity. This is even more relevant in the era of rapidly evolving radiation techniques. An ability to account for uncertainties encountered in clinical practice, including cardio‐respiratory motion, may also enable more personalised cardiac dose calculations and optimisation of radiation treatment plans. This will enable a future of individualised management, including optimisation of baseline cardiac risks, heart doses and better‐informed decisions regarding cardiac risk and monitoring.

## Data Availability

Data sharing is not applicable to this article as no new data were created or analysed in this study.
